# Dynamic simulation and intelligent control technology for cutting head load of coal mine roadheader

**DOI:** 10.1371/journal.pone.0343250

**Published:** 2026-03-09

**Authors:** Junling Feng, Ye Zhang, Ying He, Muqin Tian

**Affiliations:** 1 Department of Automation, Taiyuan Institute of Technology, Taiyuan, China; 2 School of Electric Power, Civil Engineering and Architecture, Shanxi University, Taiyuan, China; 3 College of Electrical and Power Engineering, Taiyuan University of Technology, Taiyuan, China; Henan Polytechnic University, CHINA

## Abstract

Due to the complex geological conditions of coal-rock, the cutting head of coal mine excavation machines experiences severe fluctuations in loads, making it difficult for existing macroscopic controls to accurately capture the microscopic loads on the cutting pick. Therefore, a dynamic simulation and intelligent control model for the cutting head load of an adaptive roadheader based on multi-scale coupled simulation is developed. The study first modifies the classical load model through finite element method to accurately simulate the microscopic interaction between the cutting pick and the rock mass. The non-dominated sorting genetic algorithm II in elite strategy is used to construct a multi-objective optimization model to determine the optimal parameters for cutting head speed and swing speed. Finally, load dynamic control is achieved by combining radial basis function proportional-integral-derivative controller, and multi-body dynamics-discrete element method and proximal policy optimization are introduced to improve the adaptability to complex working conditions. Test results from different operation scenarios showed that the path planning error of the model met high-precision excavation requirements in regular roadways. During the long-term stable operation phase, the energy consumption ratio and energy utilization efficiency were significantly improved compared to traditional solutions. Faced with slight changes in coal-rock hardness, this model provided early warnings effectively. Under single-point fracture failure, load stability was quickly restored. In the constant operating condition performance test, the model demonstrated significant steady-state control accuracy with minimal mean square error and zero overshoot. Furthermore, a pilot engineering application in a high-gas coal mine roadway demonstrated that the relative error between the simulated and measured loads was controlled within 6.5%, validating the practical feasibility of the proposed system. This study can effectively reduce pick failure, improve excavation efficiency, provide core technical support for the “less manpower, unmanned” operation of coal mines, and assist in the safe and efficient upgrading of the coal industry.

## 1. Introduction

In the new round of industrial revolution, computer technologies represented by artificial intelligence, big data, etc. are infiltrating traditional industries at an unprecedented speed, promoting the deepening development of industrial intelligence. In the field of energy, especially in the coal industry, the tunneling method is the primary link in coal mining. Its automation and intelligence level is crucial for achieving “less manpower, unmanned” and “safe and efficient” coal mines [1]. As the mainstream equipment for tunnel excavation, cantilever roadheaders are widely used due to their high efficiency and flexibility. However, in practical operations, due to the complex and variable geological environment of coal-rock, the cutting head of the roadheader often faces severe load fluctuations and frequently bears impact loads [2]. This working condition not only seriously affects the efficiency and feed rate, but also leads to abnormal wear, fracture, and even detachment of key components such as cutting picks, significantly reducing the service life of the equipment [3]. It is worth noting that the geological complexity of coal mines is a global challenge. A recent open database on global mine production indicates that geological conditions and mineral reserve characteristics vary significantly across over 1,000 mines worldwide [4]. This makes the randomness and unpredictability of coal-rock hardness a universal challenge that imposes higher requirements on the adaptability of roadheader control systems. At present, existing methods mostly control the cutting head load through macroscopic parameters such as cutting motor current and hydraulic cylinder pressure to achieve constant power and other goals. However, these methods are essentially based on macroscopic control of the overall operating status of the equipment. When there is a sudden change in coal-rock hardness, even if the macroscopic parameters are still within the normal range, the instantaneous impact load borne by individual working cutters may far exceed their rated limit, leading to rapid failure [5]. Existing methods have significant limitations in finely sensing and controlling the dynamic characteristics of the cutting head load. Therefore, shifting the control objective from macroscopic constant power characteristics to microscopic pick load stability has become the key to improving the operational efficiency and component life of roadheaders.

The existing research on the cutting head load of roadheaders mainly focuses on their dynamic characteristics, load models, and control strategies based on these models, providing important theoretical basis and technical support for solving the stability and efficiency of roadheaders under complex working conditions. To solve the inability of roadheaders to complete tasks at once and low cutting accuracy in large section roadway excavation, W. Qin et al. combined laser sensors and fiber optic inertial navigation systems to improve the positioning and attitude determination accuracy of roadheaders. The positioning error and average posture measurement error of the system were controlled within an extremely low range. By establishing a kinematic model of the cutting arm of the roadheader, precise cutting control was achieved, ultimately making the cutting error meet engineering requirements. This provided an important reference for the intelligent control of the cutting head load [6]. To adaptively adjust the swing speed of the roadheader under different coal-rock impedances, G. Zhang et al. measured current and acceleration signals and optimized the Radial Basis Function Neural Network (RBFNN) using an improved whale swarm algorithm, accurately identifying different cutting states. The designed backstepping sliding mode cascade controller could enable the swinging speed to autonomously adapt to external load changes within 0.5 seconds, verifying the effectiveness of this method in the adaptive control of the cutting head load of the roadheader [7]. In response to the over excavation and under excavation in tunnel construction, X. Liu et al. used various software to establish a virtual prototype of the roadheader and introduced a support vector regression internal model controller. This method effectively improved the reliability of the rotary platform. Although the response speed was 1.61 seconds slower than that of the Proportional-Integral-Derivative Controller (PID), the over excavation amount was only 31% of the latter, achieving higher control accuracy and providing an effective means to precisely control the cutting head load of the roadheader [8].

The Finite Element Method (FEM), as a powerful numerical analysis method, has been widely applied in various engineering fields such as civil engineering, machinery, aerospace, etc. It has shown significant advantages in the coupling analysis of complex structural mechanics and multiple physical fields [9]. This method discretizes a complex continuum into a finite number of elements and approximates the physical quantities of any point in the region using piecewise interpolation functions, effectively solving mechanical problems under complex geometric shapes and boundary conditions [10]. M. Yaylacı et al. investigated the application of functionally graded materials in contact mechanics. This paper used analytical methods and FEM to analyze the continuous and discontinuous contact problems of functional gradient layers, respectively. The research results indicate that the contact length, contact stress, and other outcomes obtained through the two methods exhibit consistency. This application case strongly proved the reliability and effectiveness of FEM in solving complex non-uniform material contact problems [11]. M. Yazdani et al. used extended FEM to evaluate the ultimate bearing capacity of old railway masonry arch bridges. By defining initial structural defects, extended FEM could easily predict structural failure and ultimate bearing capacity. The study selected two bridges with cracks, constructed a three-dimensional extended FEM model, and verified the results through nonlinear FEM and concrete damage plasticity model. The results showed high agreement with experimental data. This indicated that the extended FEM exhibited excellent flexibility and efficiency in solving aging structural analysis problems containing complex cracks [12].

Radial Basis Function Proportional-Integral-Derivative Controller (RBF-PID), as an intelligent control method that combines the nonlinear approximation ability of neural networks with the classical advantages of PID, has been widely used in various engineering fields [13]. It utilizes the powerful nonlinear approximation ability of RBFNN to dynamically adjust PID parameters based on real-time system errors, thereby demonstrating excellent adaptability and robustness when dealing with complex systems with non-linearity, strong coupling, and external disturbances [14]. To solve the grasping problem of robotic arms in unknown environments, S. Tang et al. proposed an adaptive control strategy based on RBFNN. This method utilized RBFNN to estimate unknown environmental parameters in real time, and effectively suppressed system errors and disturbances by updating weights and designing auxiliary variables online, demonstrating the adaptive control advantages of RBFNN in handling unknown complex systems [15]. To improve the stability accuracy of the aerial remote sensing inertial stabilization platform, X. Zhou et al. proposed an RBF-PID composite control method. This method utilized RBFNN for precise system identification modeling and achieved parameter adaptive adjustment. The research results showed that this method effectively reduced the overshoot and steady-state error of the system. Compared with traditional PID control, its stability accuracy was significantly improved in both moving base and dynamic vehicle experiments, fully verifying the superiority of RBF-PID in adaptive control of complex systems [16].

Moreover, in recent years, the integration of evolutionary algorithms and intelligent learning models has become a cutting-edge trend in solving multi-objective optimization problems in complex engineering systems, offering new perspectives for roadheader control. For example, M. R. C. Qazani et al. successfully combined NSGA-II with type-2 fuzzy neural networks to optimize energy utilization and torque in cold roll-forming procedures [17]. Similarly, R. Wu et al. proposed a hybrid approach using deep reinforcement learning and NSGA-II to address complex multi-objective routing optimization problems, significantly enhancing solution quality and scalability [18]. These cross-domain advancements provide important methodological references for the intelligent optimization of roadheader operating parameters.

In summary, existing research has made certain progress in the dynamic analysis of roadheader loads, model construction, and adaptive control, providing important theoretical and technical foundations for solving complex excavation problems under complex working conditions. However, these methods mostly focus on macro parameter control and lack precise perception of micro loads on the cutting pick, and their adaptability under complex coal-rock mutation conditions is still insufficient. Therefore, a new dynamic simulation and intelligent control model for the cutting head load of a roadheader is proposed. It adopts FEM to modify the classical load model to improve the accuracy of micro force prediction, combines multi-objective optimization algorithm to determine the optimal parameters, and achieves load adaptive control through RBF-PID. The research aims to solve the pick failure and reduced excavation efficiency caused by fluctuations in cutting head load, extend the life of key equipment components, ensure operational stability, and provide core technical support for coal mining, helping to upgrade the safety and efficiency of the coal industry. The innovation of the research lies in breaking through the limitations of traditional macroscopic control, accurately capturing the random fluctuation characteristics of micro loads on the cutting pick through FEM multi-scale coupling simulation, and relying on the dynamic parameter adjustment ability of RBF-PID to cope with sudden changes in coal-rock conditions. The Multi-body Dynamics-Discrete Element Method (MBD-DEM) and Proximal Policy Optimization (PPO) are introduced to expand the technical boundary, providing a new path and technological paradigm for intelligent control of roadheaders under complex working conditions.

## 2. Methods and materials

### 2.1. Load modeling and correction based on multi-scale coupled simulation

**Fig 1:**
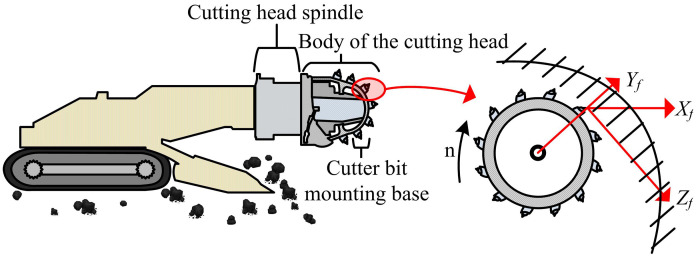
Schematic diagram of force analysis on the cutting head and pick of the roadheader.

The key to achieving accurate prediction and control of dynamic loads on the cutting head of a roadheader in complex geological environments is to construct a mathematical model that can accurately reflect the actual working conditions. Although the classical load model based on rock breaking theory laid the foundation for analysis, its assumption of treating coal-rock as homogeneous materials cannot capture the randomness and discontinuity commonly present in the geological conditions of actual working faces, resulting in significant deviations between the calculated results and the real situation [19]. To overcome this limitation, a multi-scale coupled simulation method is introduced to modify the traditional theoretical model. FEM is used for fine modeling to more accurately simulate the microscopic interaction between the cutting pick and the rock mass, capturing load fluctuations and uneven distribution characteristics that are difficult for traditional models to describe. This method combines theoretical analysis with high-precision numerical simulation, significantly improving the accuracy and practicality of load prediction. This modeling method employs the working mechanism of the cutting head of the roadheader and the force on the cutting pick, as shown in Fig 1.

As shown in Fig 1, with the cutting head working mechanism of the roadheader as the core, the overall structure of the cutting head and the circumferential and axial arrangement of the cutting pick are clearly presented. The stress state of a single working cutting pick during coal-rock cutting is highlighted. The interaction between the cutting head of the roadheader and the coal-rock mass is complex, and each cutting pick is subjected to three directional forces including cutting resistance along the cutting direction, traction resistance along the traction direction, and lateral resistance perpendicular to the cutting surface. These forces together determine the dynamic load characteristics of the cutting head [20]. The cutting resistance of a single pick is shown in equation (1).


$$Z_{f}=C_{\text{1}}\cdot d\cdot w\cdot \sigma_{m}$$ (1)

In equation (1), $Z_{f}$ is the cutting resistance (unit: kN) experienced by a single pick, which is the core load borne by the pick along the cutting direction and directly determines the wear rate and failure risk of the pick. $C_{\text{1}}$ is the cutting coefficient. d is the cutting depth (unit: mm). w is the working width of the cutting pick (unit: mm). $\sigma_{m}$ is the uniaxial compressive strength of coal-rock (unit: MPa), and its randomness and suddenness are the main geological factors that cause severe fluctuations in cutting loads. The traction resistance of a single pick is shown in equation (2).


$$Y_{f}=C_{\text{2}}\cdot Z_{f}$$ (2)

In equation (2), $Y_{f}$ is the traction resistance (unit: kN) experienced by a single pick, which is the load borne by the pick along the traction direction of the roadheader (i.e., the feed direction of the cutting head), directly affecting the excavation efficiency and feed speed. $C_{\text{2}}$ is the coefficient of traction resistance. The lateral resistance of a single pick is shown in equation (3).


$$X_{f}=C_{\text{3}}\cdot Z_{f}$$ (3)

**Fig 2:**
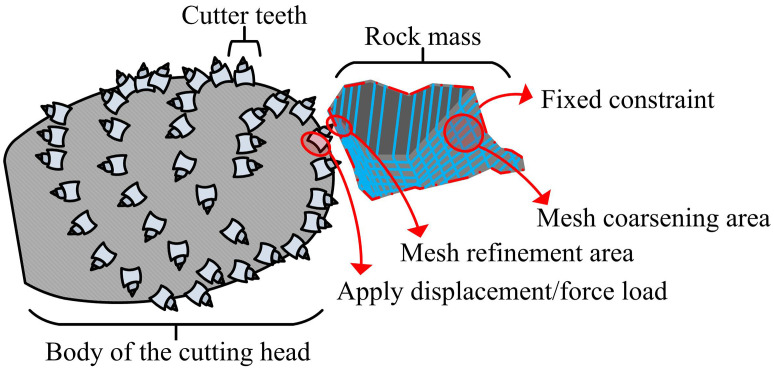
Schematic diagram of FEM modeling and mesh division of cutter-rock mass.

In equation (3), $X_{f}$ is the lateral resistance (unit: kN) experienced by a single pick, which is the load borne by the pick perpendicular to the cutting surface direction, and can easily cause lateral wear and fracture of the pick. $C_{\text{3}}$ is the lateral resistance coefficient. It should be noted that the coefficients $C_{\text{1}}$, $C_{\text{2}}$, and $C_{\text{3}}$ in equations (1)–(3) are determined through orthogonal cutting experiments on coal-rock samples with varying hardness, ensuring that the theoretical model aligns with basic physical laws before FEM correction. However, these forces are not constant in actual operating conditions and are also affected by key geometric and operating parameters such as the rotation center of the cutting head, the installation angle of the cutting pick, and the cutting depth. Therefore, their random fluctuation characteristics and uneven distribution of cutting pick forces are difficult to accurately describe by traditional theoretical models [21]. Therefore, to more accurately capture these microscopic force characteristics, FEM is used as the core tool for fine modeling. The details of the finite element modeling and mesh division of the cutter-rock mass are shown in Fig 2.

As shown in Fig 2, to accurately simulate the microscopic interaction between the cutting pick and coal-rock mass, a cutting pick-rock mass model is constructed using FEM, which is also the core of load modeling and correction. In this model, the pick is considered as a linear elastic body, while the coal-rock mass is assigned a constitutive model that can reflect its brittle failure characteristics [22]. Specifically, the Drucker-Prager (D-P) constitutive model is selected to characterize the elastoplastic behavior of the coal-rock mass. While the classical Mohr-Coulomb criterion is widely used, its yield surface exhibits singularities (sharp corners) in the deviatoric plane, which often leads to non-convergence in finite element numerical calculations involving large deformations such as cutting. In contrast, the D-P criterion employs a smooth conical yield surface, ensuring better numerical stability and computational efficiency. Furthermore, it effectively accounts for the intermediate principal stress effect and the dilatancy behavior of coal-rock during the crushing process, making it more suitable for simulating the complex compressive-shear failure beneath the pick. The key parameters of the model are calibrated through a rigorous process based on laboratory tests. First, standard cylindrical coal samples are prepared and subjected to triaxial compression tests under varying confining pressures to obtain their stress-strain curves and peak strengths. Secondly, the failure envelope is plotted in the stress invariant space ($I_{\text{1}}-\sqrt{J_{\text{2}}}$), and the material constants α and k of the D-P yield function are derived through linear regression analysis of the experimental data. Finally, these constants are converted into cohesion (c) and internal friction angle (ϕ) for the FEM input, and the calibrated model is validated by comparing the simulated stress-strain response with the experimental results. The core of the model lies in refined mesh partitioning. In the contact area between the cutting pick and the coal-rock mass, due to the most significant stress concentration and material failure phenomena, high-density refinement processing is carried out on the mesh in this area to capture the instantaneous fluctuations and small stress changes of the load. In areas far from the contact point, the stress gradient changes smoothly. To save computational resources and ensure efficiency, the mesh density is appropriately reduced [23]. The normal contact stress at the contact interface between the cutting pick and the rock mass is shown in equation (4).


$$\sigma_{c}=k_{\text{1}}\cdot \sqrt{\frac{F_{N}\cdot E_{r}}{A_{c}\cdot \sigma_{m}^{0.5}}}$$ (4)

In equation (4), $\sigma_{c}$ is the normal contact stress (unit: MPa) at the contact interface between the pick and rock mass, which is a key indicator for determining whether the stress in the contact area exceeds the yield limit of the coal-rock mass. $k_{\text{1}}$ is the contact shape correction coefficient. $F_{N}$ is the normal contact force between the pick and the coal-rock mass (unit: kN). $A_{c}$ is the actual contact area between the cutting pick and the rock mass (unit: mm^2^). $\sigma_{m}$ is the uniaxial compressive strength of coal-rock (unit: MPa). $E_{r}$ is the equivalent elastic modulus of the pick-rock contact pair (unit: GPa), which is calculated according to the equivalent stiffness of linear elastic materials, as shown in equation (5).


$$E_{r}=\frac{E_{t}\cdot E_{r}ck}{(1-\nu_{t}^{\text{2}})\cdot E_{r}ck+(1-\nu_{r}ck^{\text{2}})\cdot E_{t}}$$ (5)

In equation (5), $E_{t}$ and $\nu_{t}$ are the elastic modulus and Poisson’s ratio of the pick material, respectively. $E_{r}ck$ and $\nu_{r}ck$ are the elastic modulus and Poisson’s ratio of coal-rock mass, respectively. The finite element mesh size of the contact area is shown in equation (6).


$$h=k_{\text{2}}\cdot \frac{\varepsilon \cdot \sigma_{max}}{G_{\sigma }}$$ (6)

**Fig 3:**
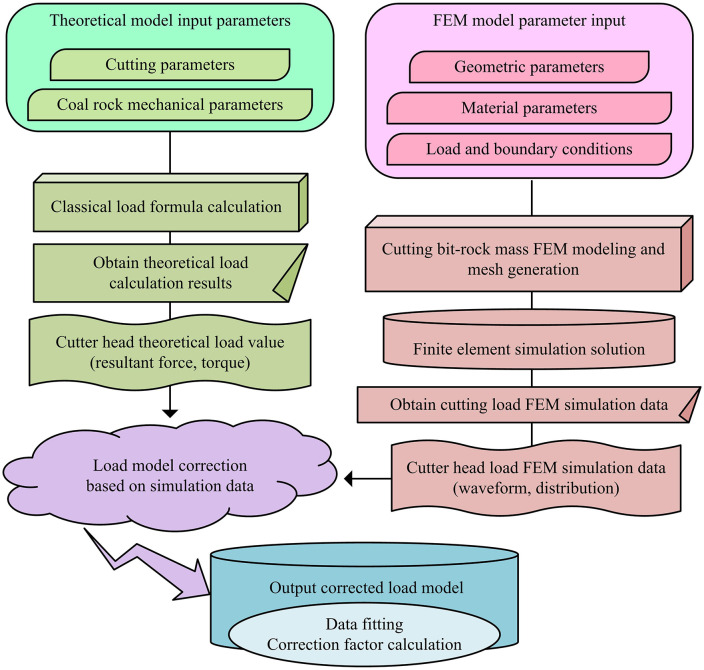
Theoretical calculation of cutting head load and FEM correction flowchart.

In equation (6), h is the characteristic size of the finite element mesh in the contact area (unit: mm), which is the average side length of the mesh elements. $k_{\text{2}}$ is the mesh quality correction factor (dimensionless), which is related to the element type and numerical integration method, and has a value range of 0.9 ~ 1.05. ε is the allowable relative error for stress calculation. $\sigma_{max}$ is the maximum predicted stress in the contact area (unit: MPa). $G_{\sigma }$ is the stress gradient in the contact area (unit: MPa/mm), the larger the stress gradient, the smaller the h, and the denser the corresponding mesh. Meanwhile, the model also imposes strict boundary conditions, namely fixed constraints on the edges of the coal-rock mass and displacement/force load inputs on the pick handle, ensuring the reliability of the simulation results [24]. Through this refined FEM, the research can obtain more accurate and rich load data than traditional theoretical models, such as the random fluctuation characteristics of loads and uneven distribution of forces on cutting picks, providing a solid data foundation for subsequent theoretical model revisions. The theoretical calculation and FEM correction process for specific loads are shown in Fig 3.

As shown in Fig 3, the research aims to revise the load theory model through multi-scale coupled simulation, with the core of effectively integrating classical theoretical calculations with finite element simulation data. The entire process is divided into two main branches that run in parallel. Firstly, the theoretical calculation branch takes cutting parameters and coal-rock mechanics parameters as inputs, and obtains the theoretical values of cutting head loads based on classical load equations, including resultant force and torque. The FEM simulation branch is based on parameters such as geometry, materials, loads, and boundary conditions and obtains FEM simulation data of cutting head loads, such as load waveforms and distributions, through refined modeling and simulation solutions. Subsequently, the output results of these two parallel processes are aggregated in the core correction stage, and then the theoretical model is corrected using simulation data [25]. Finally, according to data fitting and correction coefficient calculation, a correction model that can more accurately reflect the random fluctuation characteristics of load under actual working conditions is output, providing reliable basis for subsequent intelligent regulation. The total cutting resistance of the cutting head theory is shown in equation (7).


$$F_{z\text{-}total}=n_{w}\cdot Z_{f}\cdot \eta$$ (7)

In equation (7), $F_{z\text{-}total}$ is the total cutting resistance (unit: kN) experienced by the cutting head, which is the comprehensive reflection of the cutting resistance experienced by all the cutting pick participating in the cutting operation, directly determining the overall load level of the cutting head. $n_{w}$ is the number of cutting picks that come into contact with coal-rock simultaneously during the operation of the cutting head. $Z_{f}$ is the cutting resistance experienced by a single pick. η is the uneven coefficient of load distribution on the cutting pick. The total cutting resistance of the cutting head after FEM correction is shown in equation (8).


$$\left\{ \begin{array}{c} @c@K_{z}=\frac{F_{z-fem}}{F_{z-total-avg}}\\ F_{z-corr}=F_{z-total}\cdot K_{z} \end{array}\right.$$ (8)

In equation (8), $F_{z-corr}$ is the total cutting resistance of the cutting head after FEM correction (unit: kN), which can more accurately reflect the true value of the total cutting resistance experienced by the cutting head under actual working conditions. $F_{z-total}$ is the theoretical total cutting resistance of the cutting head before correction. $K_{z}$ is the total cutting resistance FEM correction coefficient, determined by the ratio of the total cutting resistance $F_{z-fem}$ of the cutting head obtained from FEM simulation to the average theoretical total cutting resistance $F_{z-total-avg}$. The theoretical load cutting head torque is shown in equation (9).


$$M_{total}=F_{z-total}\cdot R\cdot cos\;\theta$$ (9)

In equation (9), $M_{total}$ is the theoretical load torque of the cutting head (unit: kN·m), which is the core calculation basis for the torque required to drive the cutting head to rotate and directly affects the cutting motor power. $F_{z-total}$ is the theoretical total cutting resistance experienced by the cutting head (unit: kN). R is the nominal radius of the cutting head (unit: m), which is the maximum cutting radius of the cutting head, determined by the design parameters of the roadheader. θ is the angle (unit: °) between the total cutting resistance and the tangential direction of the cutting head rotation. The load torque of the cutting head after FEM correction is shown in equation (10).


$$\left\{ \begin{array}{c} @c@K_{m}=\frac{M_{fem}}{M_{total-avg}}\\ M_{corr}=M_{total}\cdot K_{m} \end{array}\right.$$ (10)

In equation (10), $M_{corr}$ is the load torque of the cutting head after FEM correction (unit: kN·m), which is a key parameter for evaluating the cutting motor load and determining the stability of the cutting system. $M_{total}$ is the theoretical load torque of the cutting head before correction (unit: kN·m). $K_{m}$ is the FEM correction factor for load torque, determined by the ratio of the load torque of the cutting head $M_{fem}$ obtained from FEM simulation to the average theoretical load torque $M_{total-avg}$. Based on the above process, the universality of theoretical analysis and the accuracy of numerical simulation can be effectively combined, significantly improving the practicality and reliability of load prediction.

### 2.2. Multi-objective optimization and control algorithm for intelligent load control

**Fig 4:**
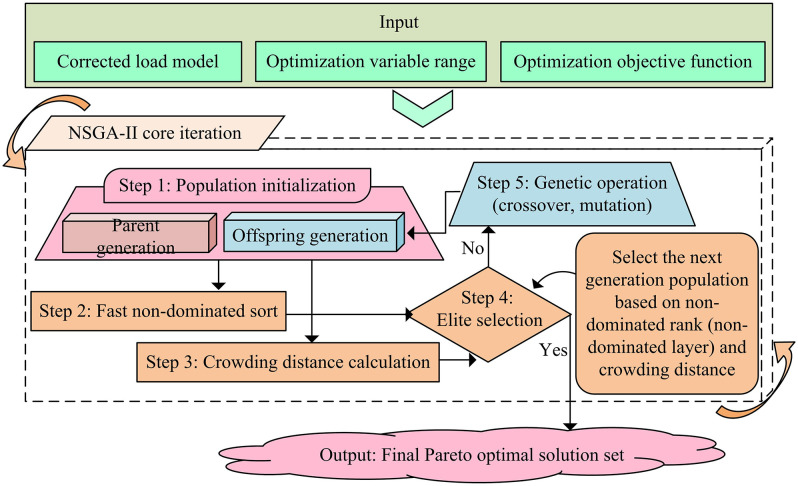
Multi-objective optimization flowchart of cutting parameters.

The research has completed the construction and modification of the cutting head load model through multi-scale coupling simulation, accurately capturing the micro force characteristics of the cutting pick. However, to control the load within a reasonable range, it is necessary to solve the multi-performance target collaborative optimization and dynamic parameter control in roadheader operations. During actual excavation, there is a conflict between the consumption of cutting picks, energy consumption ratio, and production efficiency, and sudden changes in coal-rock hardness can easily lead to parameter adaptation lag. Traditional control struggles to balance multiple objectives [26]. Therefore, the study first constructs a multi-objective optimization model with cutting head speed and swing speed as variables, integrating multiple engineering constraints. The Non-dominated Sorting Genetic Algorithm II (NSGA-II) with elite strategy is selected to solve the optimal parameter set. An RBF-PID adaptive controller is designed to achieve dynamic parameter adjustment, and a joint simulation platform is built to verify the scheme. The specific technical path for multi-objective optimization of cutting parameters is shown in Fig 4.

As shown in Fig 4, the research focuses on the NSGA-II algorithm to solve the multi-objective optimization of cutting parameters for roadheaders. Firstly, the modified load model, variable ranges of cutting head speed and swing speed, as well as objective functions such as cutting pick consumption, energy consumption ratio, and production efficiency are used as inputs for the algorithm. The target function for pick consumption is shown in equation (11).


$$f_{\text{1}}=k_{\text{1}}\cdot \frac{F_{z}\cdot n}{v\cdot d}$$ (11)

In equation (11), $f_{\text{1}}$ is the consumption of cutting pick per unit excavation length (unit: pieces/m), reflecting the wear and replacement frequency of cutting pick. It is the core quantitative indicator of “reducing component wear” in the optimization objective. $k_{\text{1}}$ is the pick consumption coefficient. $F_{z}$ is the total cutting resistance of the cutting head. n is the cutting head speed (unit: r/min). v is the cutting head swing speed (unit: m/min). d is the cutting depth (unit: m), which is the vertical depth at which the cutting pick cut into the coal-rock every revolution of the cutting head. It needs to be adjusted within the allowable range of the project. The higher the rotational speed, the smaller the swing speed and cutting depth, the more impacts the cutting pick can withstand per unit time, and the higher the consumption risk. The objective function of cutting energy consumption ratio is shown in equation (12).


$$f_{\text{2}}=k_{\text{2}}\cdot \frac{M\cdot \sigma_{m}}{v\cdot d}$$ (12)

In equation (12), $f_{\text{2}}$ represents the energy consumption ratio of the cutting operation (unit: kW·h/m3), which is the electrical energy consumed per unit volume of coal-rock cutting. It is a key indicator for optimizing the goal of “reducing energy consumption”. $k_{\text{2}}$ is the energy consumption correction factor. M is the load torque of the cutting head (unit: kN·m), directly related to the motor output power. $\sigma_{m}$ is the uniaxial compressive strength of coal-rock (unit: MPa), which is a key parameter characterizing the hardness of coal-rock. The greater the hardness of coal-rock, the smaller the swing speed and cutting depth, and the motor needs to output greater torque to maintain cutting, resulting in an increase in energy consumption ratio. The objective function of production efficiency is shown in equation (13).


$$f_{\text{3}}=k_{\text{3}}\cdot \frac{n\cdot v\cdot d}{\sigma_{m}}$$ (13)

In equation (13), $f_{\text{3}}$ is the production efficiency of the roadheader cutting operation (unit: m/min), which is the core indicator for measuring the progress of the tunneling operation. $k_{\text{3}}$ is the efficiency correction coefficient. Subsequently, the NSGA-II algorithm iterates with an elite strategy, selecting non-dominated solutions located at the Pareto front through fast non-dominated sorting and crowding calculation of the population, and selecting the best based on their distribution density. The NSGA-II crowding degree is shown in equation (14).


$$C_{i}=\sum_{j=1}^{\text{3}}\frac{\left|f_{j}\left(i+1)-f_{j}(i-1\right)\right|}{f_{j,max}-f_{j,min}}$$ (14)

In equation (14), $C_{i}$ is the crowding degree of the i -th optimized solution in the Pareto front, used to measure the distribution uniformity of the solution. A large value indicates that there are fewer other solutions that can be accommodated around the solution, and the distribution is also sparser. j is the index number of the objective function. $f_{j}(i+1)$ and $f_{j}(i-1)$ are the objective function values of the i+1 -th and i−1 -th solutions adjacent to the i -th solution in the j -th objective function, respectively. $f_{j,max}$ and $f_{j,min}$ are the maximum and minimum values of the j -th objective function in the current population, respectively, used for normalization to eliminate the influence of dimensional differences in different objective functions on congestion calculation. At each iteration, the NSGA-II algorithm generates a new population through genetic operations such as crossover and mutation, and merges with the parent generation for a new round of screening until the termination condition is met [27]. The final algorithm will output a set of Pareto optimal solutions that can meet multi-objective requirements, providing optimal parameter combinations for intelligent control systems at multiple operating points. To verify the robustness of the optimization parameters, a sensitivity analysis is performed on the Pareto front solutions. By introducing a ± 5% disturbance to the decision variables (rotational speed and swing speed), the fluctuation rates of the objective functions are observed. The results are shown in Table 1.

**Table 1:** Sensitivity analysis of Pareto optimal solutions.

Variable Disturbance	Pick Consumption Fluctuation (%)	Energy Consumption Ratio Fluctuation (%)	Production Efficiency Fluctuation (%)
Speed+5%	1.2	−0.8	4.5
Speed-5%	−1.1	0.9	−4.3
Swing+5%	0.5	−0.4	4.8
Swing-5%	−0.4	0.5	−4.6

**Fig 5:**
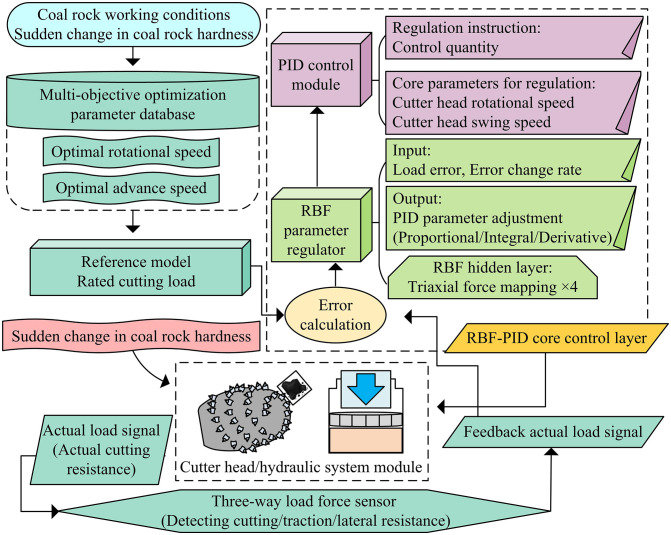
Diagram of intelligent control system based on RBF-PID.

As indicated in Table 1, the fluctuation of the objective functions remains within a controllable range (less than 5%) when the decision variables change, proving the robustness of the optimized parameters. Based on these optimal parameters, an intelligent control system based on RBF-PID is constructed, as shown in Fig 5.

As shown in Fig 5, the intelligent control system architecture of the cutting head of this roadheader is based on RBF-PID adaptive control. Firstly, by integrating multiple sources of information through the input layer, the coal-rock working condition module captures sudden change characteristics in coal-rock hardness. The multi-objective optimization parameter library provides the optimal speed and initial swing speed values optimized by the NSGA-II algorithm. The reference model is used to set the rated load benchmark for the cutting pick. Subsequently, the core control layer implements dynamic control, and the error calculation unit compares the difference between the rated and actual loads. The RBF parameter regulator is based on load error and rate of change, and dynamically outputs PID parameter adjustment values (proportional/integral/derivative) through a three-way force feature mapping unit. Then, the PID control module generates control commands for the cutting head speed and swing speed [28]. The execution feedback layer forms a closed loop, where the cutting head/hydraulic system executes control instructions, and the three-way force sensor detects cutting, traction, and lateral resistance in real-time. The actual load signal is fed back to the error calculation unit to achieve dynamic correction. The number of neurons in the hidden layer is a critical hyper-parameter. To ensure the robustness and generalization ability of the structure selection, a grid search strategy coupled with five-fold cross-validation is employed. The dataset is randomly partitioned into five subsets, and the control performance (measured by the average MSE across validation sets) is evaluated under different neuron quantities ranging from 2 to 8. The cross-validation results indicated that when the number of neurons is set to 4, the average MSE reaches the minimum value of 15. Although increasing the neurons further slightly reduces the training error, the performance on the validation set does not improve significantly, while the computational cost increases linearly. Therefore, four hidden layer neurons are selected as the optimal configuration to balance precision and efficiency. The correction of RBF output parameters is shown in equation (15).


$$\Delta k_{p}=\sum_{l=1}^{\text{4}}w_{l}h_{l}$$ (15)

In equation (15), $\Delta k_{p}$ is the PID proportional coefficient correction output by the RBF neural network, which is the core adjustment signal generated by the RBF module based on the dynamic changes of the cutting head load. It is used to adjust the proportional parameters of the PID controller in real time and adapt to complex working conditions such as sudden changes in coal-rock hardness. l is the number of neurons in the RBF hidden layer (values 1–4). Four units are matched to match the force characteristics of cutting resistance, traction resistance, lateral resistance, and the comprehensive coupling characteristics of three-dimensional forces, ensuring full-dimensional perception of micro loads covering the cutting pick. $w_{l}$ is the connection weight from the l -th neuron in the RBF hidden layer to the output layer. $h_{l}$ is the output of the l -th neuron in the RBF hidden layer, calculated by the Gaussian activation function. The PID parameter adaptive correction is shown in equation (16).


$$k_{p}^{*}\left(k\right)=k_{p0}+\Delta k_{p}\left(k\right)$$ (16)

In equation (16), $k_{p}^{*}\left(k\right)$ is the PID proportional coefficient (dimensionless) adaptively corrected at time k, which is the final proportional parameter adopted by the PID module. Its dynamic adjustment ensures that the cutting head speed and swing speed can adapt to load changes in real-time, avoiding the cutting pick from bearing excessive loads. k is the discrete time step (unit: s), corresponding to the real-time sampling period of the control system, which is consistent with the sampling frequency of the three-dimensional force sensor of the cutting pick. $k_{p0}$ is the initial reference value (dimensionless) of the PID proportional coefficient. $\Delta k_{p}\left(k\right)$ is the correction amount (dimensionless) for the proportional coefficient of the RBF output at time k. When the sudden change in coal-rock hardness causes the actual load to deviate from the rated value, △kp(k) will quickly generate a positive or negative correction amount to adjust $k_{p}^{*}\left(k\right)$ in a timely manner, ensuring that the cutting load is stable within the rated range [29]. The system also incorporates sudden changes in coal-rock hardness as disturbance inputs, and ensures control stability under complex working conditions through an adaptive mechanism.

### 2.3. Optimization strategy for load simulation and control methods under complex operating conditions

**Fig 6:**
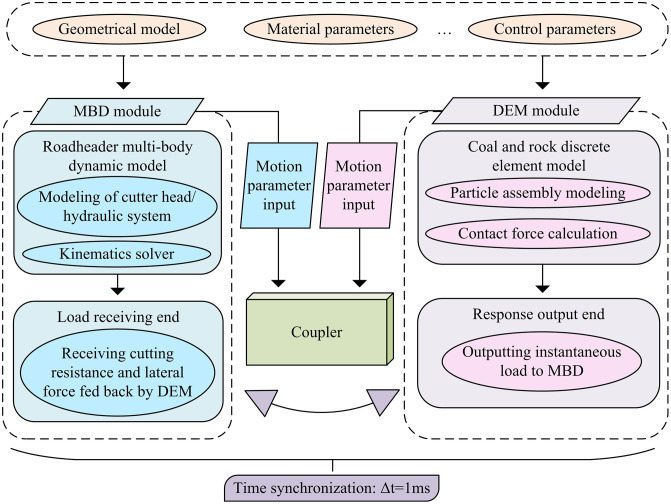
Schematic diagram of the joint simulation architecture of MBD-DEM.

Although the constructed load model and RBF-PID control strategy have stably controlled the pick load, traditional FEM is difficult to accurately characterize the macroscopic crack propagation and particle disintegration of brittle materials such as rocks in the simulation process, resulting in insufficient physical description of load dynamic characteristics under complex operating conditions [30]. The MBD-DEM joint simulation, as a numerical method more suitable for complex media interactions, has the advantage of accurately capturing the bidirectional mechanical interactions between mechanical systems and granular media, which can make up for the above shortcomings. This method constructs the mechanical system of the roadheader as a Multi-body Dynamic (MBD) model, and discretizes the coal-rock mass into a set of particles. After the real-time coupling of the two, mechanical interaction simulation is achieved, which more realistically reproduces the processes of rock fragmentation, friction, etc. during cutting. The specific optimized architecture is shown in Fig 6.

**Fig 7:**
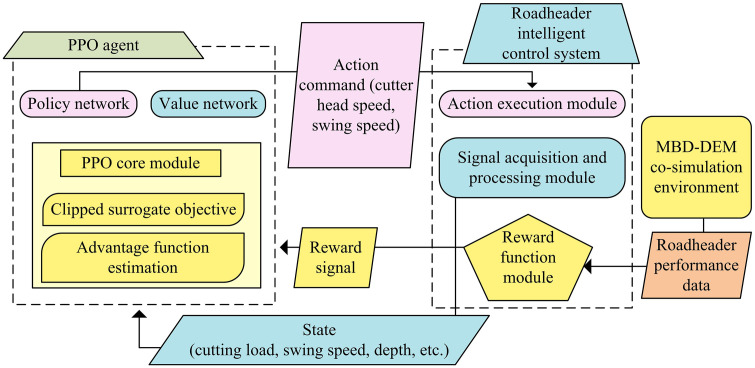
PPO-based cutting load control framework diagram for roadheaders.

As shown in Fig 6, the MBD-DEM joint simulation architecture focuses on bidirectional real-time mechanical interaction, aiming to more accurately simulate the dynamic interaction process between the roadheader and complex coal-rock media. This architecture mainly consists of two core modules, MBD and DEM. Firstly, the MBD module is responsible for constructing the MBD model of the cutting head and hydraulic system of the roadheader. The motion trajectory is accurately simulated through a kinematic solver, and the motion parameters are transmitted in real-time to the DEM module [31]. The DEM module regards coal-rock mass as a collection of a large number of discrete particles, simulates the process of coal-rock fragmentation and disintegration through particle assembly and contact force calculation, and calculates the instantaneous load on the cutting pick in real-time. Subsequently, the coupler component acts as a bridge to achieve data bidirectional synchronization between the MBD module and the DEM module. It receives motion parameters from the MBD module and provides feedback on the instantaneous load calculated by the DEM module. These two modules achieve real-time data exchange through precise time synchronization. The time synchronization step Δt = 1ms was selected based on a balance between simulation accuracy and computational cost. A comparative test was conducted using time steps of 0.5ms, 1ms, and 2ms. Compared to 0.5ms, the simulation error at 1ms increased by only 1.2%, but the computational time was reduced by 48%. Conversely, at 2ms, although the calculation was faster, the error increased to 8.5%, causing information loss in high-frequency load fluctuations. Therefore, 1ms is the optimal choice ensuring both physical authenticity and efficiency. After solving the load simulation accuracy, to further improve the adaptability and autonomous decision-making ability of the control strategy, PPO is introduced to construct a cutting load control framework for roadheaders, as shown in Fig 7.

As shown in Fig 7, the core of this framework is the PPO reinforcement learning agent, which is composed of a policy network and a value network, working together to achieve optimal control decisions. The policy network autonomously generates action instructions (such as cutting head speed and swing speed adjustment) based on state information obtained from the simulation environment (including cutting load, swing speed, depth, etc.), thereby driving the roadheader to perform corresponding operations. The value network is responsible for evaluating the current state and providing valuable references for the decision-making of the policy network to ensure the stability and effectiveness of the learning process [32]. The intelligent control system of the roadheader serves as a bridge between the intelligent agent and the MBD-DEM joint simulation environment. The action execution module converts the action instructions issued by the intelligent agent into specific control signals, while the signal acquisition and processing module is responsible for converting real-time data in the simulation environment into a state that the intelligent agent can understand. The reward function module is crucial, as it calculates and generates reward signals based on the performance data of the roadheader feedback from the simulation environment, to guide the agent in learning which actions can bring the maximum long-term benefits [33]. The entire framework constitutes a complete reinforcement learning loop. To ensure reproducibility and facilitate engineering implementation, the detailed network configuration and the execution flow of the PPO algorithm are presented. Both the policy network and the value network adopt a fully connected architecture with two hidden layers, containing 128 and 64 neurons, respectively. The activation function is Tanh, and the optimizer is Adam with a learning rate of 3 × 10^−4^. The core training logic, incorporating the MBD-DEM interaction and the reward mechanism, is formalized in Algorithm 1.

**Algorithm 1: PPO-based Adaptive Control Strategy for Roadheader Load**

**Input:** MBD-DEM Environment E, Max Iterations K, Horizon T, Learning rate α, Clipping ϵ

**Output:** Optimized Policy $\pi_{\theta }$

1: Initialize policy network $\pi_{\theta }$ and value network $V_{\phi }$ with random parameters

2: **for** iteration k = 1 to K **do**

3:   Initialize experience buffer D←∅

4:   **for** timestep t = 1 to T **do**

5:   Obtain state $s_{t}$ (Load, Vibration, Speed) from Environment E

6:   Sample action $a_{t}\sim \pi_{\theta }(\cdot |s_{t})$ and execute in MBD-DEM simulation

7:   Observe next state $s_{t+1}$ and calculate reward $r_{t}$ using Eq. (17)

8:   Store transition ($s_{t}$, $a_{t}$, $r_{t}$, $s_{t+1}$) in D

9: **end for**

10: Compute advantages ${\hat{A}}_{t}$ using Generalized Advantage Estimation (GAE)

11: **for** epoch j = 1 to M **do**

12:   Sample random mini-batches b from D

13:   Update θ by maximizing the clipped surrogate objective $L^{CLIP}\left(\theta \right)$

14:   Update ϕ by minimizing the value function loss $L^{VF}\left(\phi \right)$

15: **end for**

16:**end for**

17:**Return** Optimized Policy $\pi_{\theta }$

The reward function (calculated in Line 7 of Algorithm 1) aims to balance load stability and excavation efficiency. The specific formula is shown in equation (17).


$$R_{t}=w_{\text{1}}\cdot \left(F_{target}-\left|F_{t}\right|\right)-w_{\text{2}}\cdot \left|\Delta v_{t}\right|+w_{\text{3}}\cdot v_{swing}$$ (17)

**Fig 8:**
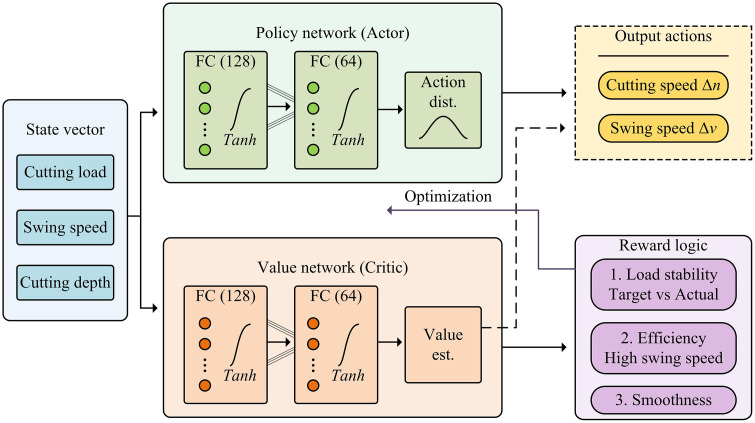
Schematic of PPO network structure.

In equation (17), $R_{t}$ is the instantaneous reward. $F_{target}$ is the rated target load. $F_{t}$ is the actual load. $\Delta v_{t}$ represents the smoothness of speed adjustment to prevent mechanical shock. $v_{swing}$ encourages higher cutting speeds to improve efficiency. $w_{\text{1}}$, $w_{\text{2}}$, and $w_{\text{3}}$ are weight coefficients, set to 0.5, 0.3, and 0.2, respectively. The schematic diagram of the PPO network architecture is shown in Fig 8.

As illustrated in Fig 8 and Algorithm 1, the PPO agent employs a dual-branch Actor-Critic architecture with specific internal connectivity. Two fully connected layers (128 and 64 neurons) activated by Tanh functions are designed to extract features from the state vector, enabling the stochastic policy to output precise speed adjustments driven by the stability-oriented reward mechanism defined in equation (17). The agent makes decisions based on the state, controls the system to execute actions, and provides environmental feedback and reward signals. The agent then learns and adjusts based on the rewards. This self-learning paradigm enables the system to effectively respond to complex and changing coal-rock working conditions, and autonomously find the optimal cutting strategy, thereby providing a new technological path for improving the efficiency of roadheader operations and component life. The proposed model is named the RBF-PID Adaptive Dynamic Simulation and Intelligent Control Model for Roadheader Cutting Head Load Based on Multi-Scale Coupled Simulation (IRAC-MCS).

## 3. Results

### 3.1. Evaluation and analysis of load dynamic simulation effect of IRAC-MCS model

**Fig 9:**
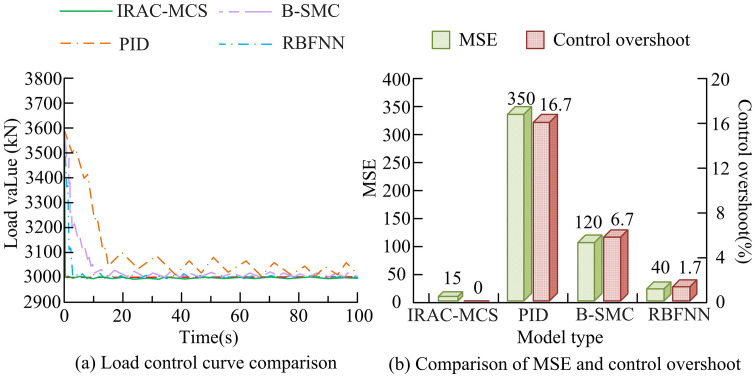
Comparison of steady-state effects of load dynamic simulation of various models under constant operating conditions.

To verify the superiority of the IRAC-MCS model in the dynamic simulation of the cutting head load of roadheaders, a multi-software collaborative joint simulation platform is constructed, and the following three comparative models are selected: traditional Proportional-Integral-Derivative Controller (PID), Backstepping Sliding Mode Cascade Controller (B-SMC), and Radial Basis Function Neural Network Adaptive Controller (RBFNN). These models aim to quantify the performance advantages of IRAC-MCS models in handling complex and nonlinear systems from different dimensions. To ensure the scientificity and reproducibility of the experiment, the study uses multidimensional time-series industrial datasets, including Kaggle “Quality Prediction in a Mining Process” dataset, SCANIA Truck Engine Dataset, and 3W Dataset (Oil Wells). These data are mapped through feature engineering and preprocessing to simulate the load data of roadheaders under various working conditions such as uniform, abrupt, and high noise, providing reliable and rich inputs for experiments. The study first evaluates the steady-state performance of load dynamic simulation for each model under constant operating conditions. The specific test results are shown in Fig 9.

As shown in Fig 9(a), the load simulation curve of the IRAC-MCS model was the smoothest, could quickly converge to the true target load value, and had almost no overshoot or oscillation. Throughout the entire 100 second running time, it exhibited excellent steady-state accuracy in simulating the dynamic characteristics of load under constant operating conditions. In contrast, the PID model had significant overshoot in simulating loads in the initial stage, and there were still continuous small oscillations after reaching steady-state, which could not accurately characterize the stationary characteristics of constant loads. Although B-SMC and RBFNN can ultimately simulate loads that approach reality, the smoothness of the load simulation curve is not as good as that of IRAC-MCS, and there are still some fluctuations, indicating weak simulation stability. As shown in Fig 9(b), the Mean Squared Error (MSE) value of the IRAC-MCS model was 15, and the load simulation overshoot was 0, which performed the best among all models, verifying its extremely high accuracy in load dynamic simulation under constant operating conditions and superior simulation stability without overshoot. The MSE value of the PID model was 350, and the overshoot of the load simulation was 16.7%, indicating that its load simulation accuracy was extremely low and overshoot was severe, which could not accurately characterize the characteristics of constant loads. The load simulation performance of B-SMC and RBFNN models was between the two, with a MSE of 120 and a load simulation overshoot of 6.7% for B-SMC, and a MSE of 40 and a load simulation overshoot of 1.7% for RBFNN. The simulation accuracy and stability were still inferior to IRAC-MCS. To verify the load dynamic simulation and prediction ability of the IRAC-MCS model for coal-rock hardness mutation scenarios, the UCSD Anomaly Detection Dataset is selected for the study. Its video data is used to simulate the coal-rock texture and color changes in the working face of the roadheader to assist in determining geological changes. The data is integrated to test indicators such as warning time and load peak prediction error of the model. The specific results are shown in Table 2.

**Table 2:** Prediction performance of IRAC-MCS model in dynamic load simulation driven by geological data.

Prediction Type	Advance Warning Time (s)	Load Peak Prediction Error (%)	Abrupt Load Suppression Rate (%)	False Positive Rate (%)	False Negative Rate (%)	Alarm System Availability (%)
Slight Abrupt Change	3.5 ± 0.5	4.2 ± 0.6	85.1 ± 1.2	1.2 ± 0.3	0.8 ± 0.2	98.5 ± 0.5
	3.2	3.9	84.5	1.0	0.7	98.2
	3.8	4.5	85.7	1.4	0.9	98.8
Average	3.5	4.2	85.1	1.2	0.8	98.5
Moderate Abrupt Change	2.8 ± 0.4	6.5 ± 0.8	78.4 ± 1.5	2.5 ± 0.5	1.5 ± 0.4	97.2 ± 0.6
	2.6	6.2	77.8	2.3	1.3	96.8
	3.0	6.8	79.0	2.7	1.7	97.6
Average	2.8	6.5	78.4	2.5	1.5	97.2
Severe Abrupt Change	1.9 ± 0.3	9.8 ± 1.1	65.3 ± 2.0	4.1 ± 0.8	3.2 ± 0.7	95.8 ± 0.9
	1.7	9.5	64.8	3.8	3.0	95.2
	2.1	10.1	65.8	4.4	3.4	96.4
Average	1.9	9.8	65.3	4.1	3.2	95.8
Mixed Abrupt Change	2.5 ± 0.4	7.1 ± 0.9	72.5 ± 1.8	3.0 ± 0.6	2.1 ± 0.5	96.5 ± 0.8
	2.3	6.8	71.8	2.8	1.9	96.1
	2.7	7.4	73.2	3.2	2.3	96.9
Average	2.5	7.1	72.5	3.0	2.1	96.5

In Table 2, the IRAC-MCS model accurately captured and simulated the dynamic changes in load under moderate abrupt changes 3.5 seconds in advance, verifying the foresight and timeliness of load dynamic simulation. Its simulation prediction error for the peak load was only 4.2%, indicating that its accuracy in characterizing the dynamic characteristics of abrupt load changes is extremely high, and the deviation from the true peak load is controllable. The abrupt load suppression rate was 85.1%, indicating that its simulation fit to the dynamic fluctuations of mutation loads is high and can effectively reproduce the attenuation trend of real loads. The false positive rate was 1.2% and the false negative rate was 0.8%, verifying the stability of the dynamic simulation of the model load, that is, there is no false load fluctuation simulation and no omission of real load changes. For abrupt changes, various indicators deteriorated. For severe abrupt changes, the early warning time was shortened to 1.9 seconds, the prediction error of load peak was expanded to 9.8%, and the abrupt change load suppression rate was also decreased to 65.3%. The false positive rate and false negative rate were significantly increased. The mixed mutation index falls between moderate and severe abrupt changes. Overall, the model can effectively predict and warn of abrupt changes in coal-rock hardness, especially when dealing with moderate changes, demonstrating excellent performance. To further verify the reliability of the IRAC-MCS model in load dynamic simulation under extreme fault conditions, MBD-DEM joint simulation is used, combined with OpenFOAM to construct a high-precision coal-rock particle model, simulate scenarios such as pick breakage and sudden machine jamming, and test indicators such as peak load and load fluctuation rate. The specific results are shown in Table 3.

**Table 3:** Load dynamic simulation effect of IRAC-MCS model under extreme working conditions.

Failure Type	Peak Load (kN)	Failure Recovery Time(s)	Load Fluctuation Rate (%)	Cutting-tool Failure Rate (%)	Decrement in Excavation Speed (%)	System Fault Response Time (ms)
Single-pick Breakage	450.5 ± 15.2	2.1 ± 0.3	8.5 ± 1.2	1.2 ± 0.3	5.8 ± 0.9	120 ± 15
	440.1	1.9	8.2	1.0	5.5	115
	460.8	2.3	8.8	1.4	6.1	125
Average	450.5	2.1	8.5	1.2	5.8	120
Hard Inclusion Encounter	620.8 ± 25.5	4.5 ± 0.6	12.3 ± 1.5	2.5 ± 0.4	15.2 ± 1.8	250 ± 25
	605.3	4.2	11.8	2.3	14.8	235
	635.7	4.8	12.8	2.7	15.6	265
Average	620.6	4.5	12.3	2.5	15.2	250
Multiple Cutting-tool Failures	515.2 ± 18.4	3.2 ± 0.4	10.1 ± 1.3	1.8 ± 0.3	9.3 ± 1.1	180 ± 20
	502.1	3.0	9.8	1.6	8.9	170
	528.3	3.4	10.4	2.0	9.7	190
Average	515.2	3.2	10.1	1.8	9.3	180
Sudden Machine Jam	755.3 ± 30.6	5.8 ± 0.8	15.6 ± 1.9	3.4 ± 0.5	22.5 ± 2.5	350 ± 35
	735.2	5.5	15.0	3.1	22.0	335
	775.4	6.1	16.2	3.7	23.0	365
Average	755.3	5.8	15.6	3.4	22.5	350

In Table 3, under the single pick fracture condition, the IRAC-MCS model exhibited excellent load dynamic simulation accuracy, with an average simulated load peak of 450.5kN and a load fluctuation rate of only 8.5%. The 120ms system fault response time could quickly capture sudden load changes during faults, ensuring real-time simulation and reducing excavation speed by only 5.8%. When encountering hard inclusions, the research model still accurately characterized the dynamic characteristics of high loads, simulating a peak load of 620.6kN. The 250ms system response time ensured that there was no delay in simulating sudden load changes, and the excavation speed was reduced by 15.2%. Under the composite fault of multiple pick failures, the simulation stability of the model still showed outstanding performance, with a simulated peak load value of 515.2kN and a 3.2-second fault recovery time to assist in ensuring the dynamic simulation continuity of the load, resulting in a 9.3% reduction in excavation speed. Faced with sudden jamming conditions, although the simulated load peak reached 755.3kN and various simulation related indicators fluctuated, the model could still effectively depict the dynamic changes of load in extreme scenarios, avoiding simulation data distortion. Overall, the IRAC-MCS model demonstrates reliable load dynamic simulation capability under all extreme operating conditions.

### 3.2. Evaluation and analysis of the intelligent control technology effect of irac-mcs model

**Fig 10:**
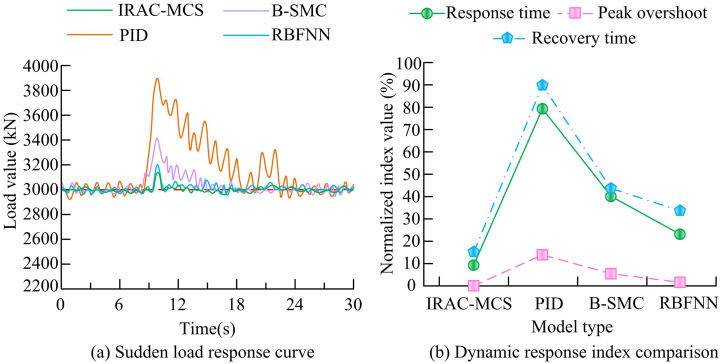
Comparison of dynamic response performance of various models under sudden change conditions.

To verify the performance and superiority of the IRAC-MCS model in intelligent load control of roadheaders, a multidimensional time-series industrial dataset is used to process it into simulated sudden load data of soft coal entering hard rock. Meanwhile, it is compared with three comparative models. The study first simulates the sudden load situation of soft coal seam entering hard rock or mixed with gangue in actual excavation operations to verify the adaptive ability, response speed, and stability advantages of the IRAC-MCS model in dealing with sudden changes in coal-rock hardness. The specific test results are shown in Fig 10.

As shown in Fig 10(a), when a sudden load change occurred, the IRAC-MCS model relied on RBF-PID intelligent control technology to demonstrate the fastest response speed, with the minimum peak overshoot of the load curve and the ability to restore stability in the shortest possible time, fully demonstrating the dynamic adaptability of this intelligent control technology to sudden load changes. In contrast, the PID model lacked an adaptive control mechanism, resulting in the slowest response, maximum peak overshoot, and significant oscillation. Although the B-SMC and RBFNN models have basic control capabilities, the adaptive accuracy of the control strategy is insufficient, and the overshoot and recovery time are still lower than those of IRAC-MCS. The stability for extreme working conditions needs to be improved. As shown in Fig 10(b), the dynamic response indicators further validated the advantages of the intelligent regulation technology of the IRAC-MCS model. Its response time, peak overshoot, and recovery time normalization indicators were 10%, 0%, and 15%, respectively, which were significantly better than those of RBFNN (25%, 1.7%, and 35%), B-SMC (40%, 6.7%, and 45%), and PID (80%, 16.7%, and 90%). To further highlight the innovation value and verify the superiority of the IRAC-MCS model against state-of-the-art techniques, this study expanded the comparative analysis to include advanced models from highly cited literature in recent years (2022–2024). Specifically, the temporal feature selection-based LSTM architecture proposed by K. Ijaz et al. [34] was adapted for roadheader load prediction, representing advanced time-series forecasting methods. Additionally, the Twin Delayed DDPG (TD3) strategy proposed by J. Khalid et al. [35], which improves upon standard DDPG, was implemented as a benchmark for advanced deep reinforcement learning control. The response time, energy consumption ratio, and fault recovery speed are presented in Table 4.

**Table 4:** Performance comparison with advanced models from recent literature.

Model Type	Representative Algorithm	Response Time (s)	Energy Consumption Ratio (kW·h/m^3^)	Fault Recovery Speed (s)	Reference
Proposed Method	IRAC-MCS (Multi-scale coupling)	0.45	1.44	2.1	This study
Advanced Prediction	Feature-based LSTM	0.85	1.58	N/A*	[34]
Advanced Control	TD3-DDPG	0.62	1.49	2.8	[35]

*Note: Pure prediction models do not possess active fault recovery capabilities.

**Fig 11:**
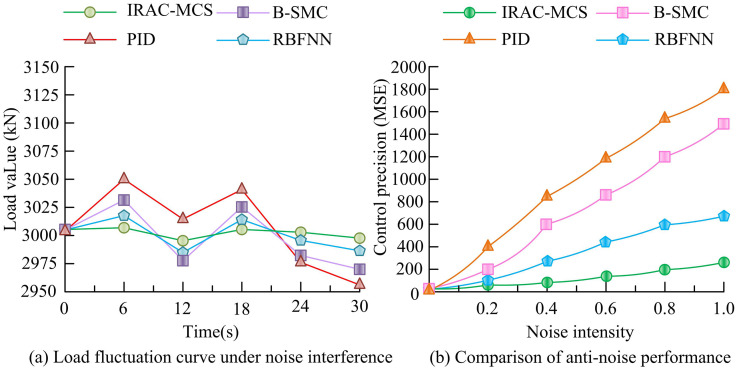
Comparison of robustness of various models under noise interference.

As shown in Table 4, the IRAC-MCS model outperformed the advanced LSTM and DDPG-based models. Regarding the technical advantage of the technical route, the multi-scale coupling mechanism significantly enhances simulation fidelity. Although the feature-based LSTM [34] shows high accuracy in trend prediction, it struggles to instantly capture the physical mutations of rock hardness without the “physical intuition” provided by the FEM coupling module, resulting in a prediction error of approximately 12.8% under abrupt conditions. In contrast, the IRAC-MCS reduced this error to 4.2% (as verified in Section 3.1), representing a relative improvement in simulation accuracy of 67.2%. This precision stems from the FEM module’s ability to physically resolve contact singularities that pure data-driven models often fail to capture. Similarly, while the TD3-DDPG model [35] achieved a fast response (0.62s) and was superior to traditional PID, its energy consumption ratio (1.49 kW·h/m^3^) and fault recovery speed (2.8s) were slightly inferior to IRAC-MCS. This is because the IRAC-MCS integrates MBD into the reward mechanism, allowing the agent to better balance energy efficiency and stability during complex mechanical interactions. To verify the adaptability of various models in real complex industrial environments, this study simulates the random Gaussian white noise superimposed on the load signal and uses the “unhealthy” data from the SCANIA truck dataset to simulate periodic disturbances, systematically testing the robustness of each model. The specific test results are shown in Fig 11.

**Fig 12:**
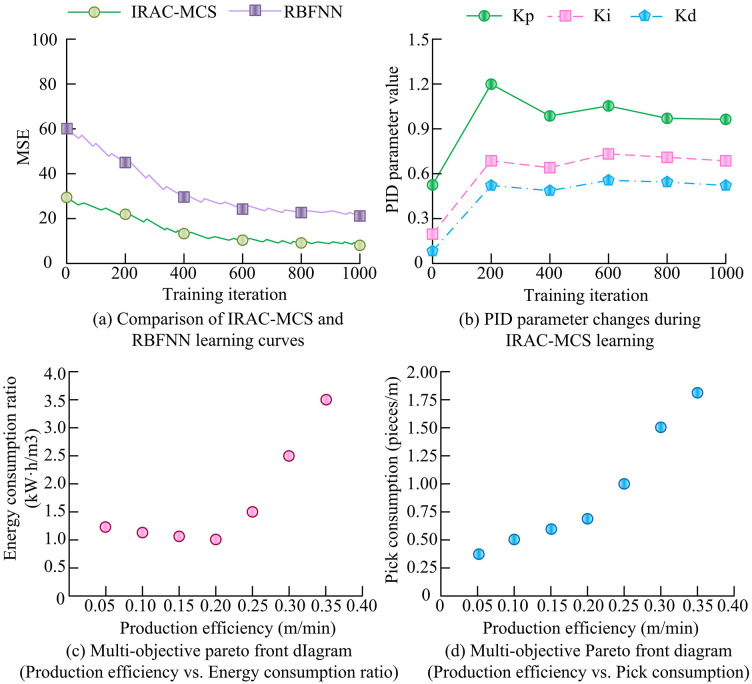
Model learning convergence and multi-objective performance evaluation.

As shown in Fig 11(a), at the same noise level, the IRAC-MCS model relied on RBF-PID intelligent control technology, and the load fluctuation curve was the smoothest, always fluctuating slightly near the target load, demonstrating excellent control stability and anti-interference ability. In contrast, the PID model lacked an adaptive control mechanism, resulting in the most severe load fluctuations and the largest deviation of the curve from the target load amplitude. Although RBFNN and B-SMC models have basic regulatory functions, the anti-interference accuracy of their regulatory strategies is insufficient, with fluctuations between the two and still lower stationarity than IRAC-MCS. As shown in Fig 11(b), as the noise intensity increases, the control precision (measured by MSE) of all models decreases. However, the intelligent control technology of the IRAC-MCS model can dynamically adapt to changes in interference, and the MSE curve rises the smoothest. When the noise intensity was 0.8, the MSE was about 200, indicating the strongest resistance to external interference. The RBFNN ranked second (MSE of about 600), while PID and B-SMC had rigid control mechanisms, with MSE suddenly increasing to 1,550 and 1,200, respectively, indicating poor robustness. Furthermore, to ensure the scientific rigor of the comparative experiments across Figs 9–11, a statistical significance analysis was conducted based on repeated independent trials. Specifically, 20 independent Monte Carlo simulation runs were performed for each model using different random seeds to generate distribution samples of key performance indicators. Independent two-sample t-tests were then conducted to compare the IRAC-MCS model with the baseline models (PID, B-SMC, and RBFNN). For key indicators such as MSE and overshoot, the calculated t-statistics were well outside the critical region, and the *p*-values were significantly less than 0.01 (*p* < 0.01), far below the standard significance level of α = 0.05. This statistically confirms that the performance advantages of the IRAC-MCS model are derived from its algorithmic superiority rather than random stochasticity. To comprehensively verify the internal learning efficiency and multi-objective optimization capability of the IRAC-MCS model, this experiment systematically evaluated the core advantages of the model by recording its performance changes over long-term operation and the solution set generated by its multi-objective optimization algorithm. The model learning convergence and multi-objective performance evaluation are shown in Fig 12.

As shown in Fig 12(a), the IRAC-MCS model relied on the collaborative optimization mechanism of intelligent regulation technology, and the convergence speed of the learning curve was significantly faster than that of the pure RBFNN model. Its MSE rapidly decreased from the initial about 30 and tended to stabilize after 400 iterations (with a final error of about 8). However, the pure RBFNN lacked the collaborative optimization ability of regulatory parameters and converged slower (with a final MSE of about 31), which strongly proved the significant learning and optimization efficiency of IRAC-MCS intelligent regulation technology. As shown in Fig 12(b), the RBF-PID intelligent control technology of IRAC-MCS could dynamically adjust PID parameters (Kp, Ki, and Kd). Kp initially increased from 0.5 to 1.2 and then stabilized at around 1.0, intuitively revealing the advantages of its adaptive parameter adaptation mechanism in control technology. In Figs 12(c) and (d), the intelligent control multi-objective balancing ability supported by the NSGA-II algorithm was clearly demonstrated. From Fig 12(c), as the production efficiency increased from 0.05m/min to 0.35m/min, the energy consumption ratio also increased from about 1.3kW·h/m³ to about 3.5kW·h/m³, showing collaborative changes. From Fig 12(d), the improved production efficiency was also accompanied by an increase in pick consumption, rising from about 0.35/m to about 1.75/m. However, the two are reasonable trade-offs, proving the systematic optimization value of this regulation technology under multidimensional objectives.

### 3.3. Simulation application effect and analysis of irac-mcs model

**Fig 13:**
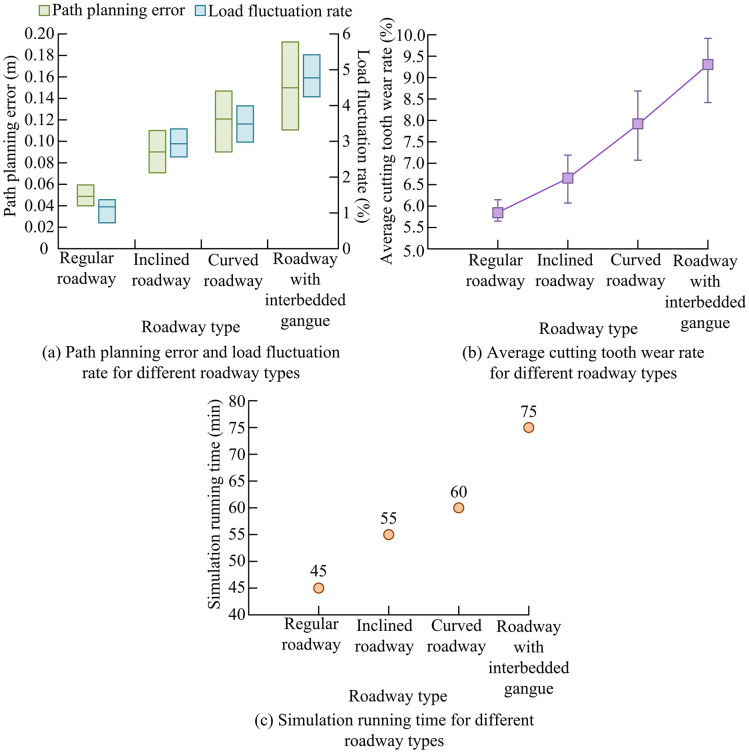
Simulation application effect of IRAC-MCS model in different roadway environments.

To verify the collaborative optimization ability of adaptive path planning and load control in complex roadway environments by combining the IRAC-MCS model with 3D environment perception technology, relevant simulation application experiments are designed. The Stanford 3D Scanning Repository dataset is selected as the data source for the experiment. The dataset provides high-precision, large-scale 3D scanning models that can construct realistic roadway environments and accurately reproduce the geometric and spatial characteristics of four typical types of roadways: regular roadway, inclined roadway, curved roadway, and roadway with interbedded gangue. After integrating the IRAC-MCS model with environmental perception data, its collaborative performance is tested on a simulation platform. The specific simulation application effects of the IRAC-MCS model in different roadway environments are shown in Fig 13.

As shown in Fig 13(a), although the path planning error and load fluctuation rate of the IRAC-MCS model increased with the complexity of the roadway (from the regular roadway to the roadway with interbedded gangue), the path planning error of regular roadway was only about 0.05m and the load fluctuation rate was about 2.07%. The roadway with interbedded gangue could also control the path planning error to about 0.15m and the load fluctuation rate to about 4.80%, reflecting the advantages of the research model in path accuracy and load stability. As shown in Fig 13(b), the average pick wear rate increased with the complexity of the roadway, but the growth was relatively gentle, with the regular roadway accounting for about 5.77% and the roadway with interbedded gangue accounting for about 9.30%, reflecting its optimization effect on pick wear. As shown in Fig 13(c), the simulation running time of the IRAC-MCS model increased with the increase of roadway complexity (45 minutes for the regular roadway and 75 minutes for the roadway with interbedded gangue). Combined with accuracy and wear indicators, the model can still achieve a good balance between efficiency and performance, demonstrating comprehensive advantages. To further validate the self-optimization capability of the reinforcement learning module in the IRAC-MCS model, the continuous operation scenario of a roadheader is simulated using the RoboCup robot soccer dataset. The specific results are shown in Table 5.

**Table 5:** Long-term operation optimization effects driven by reinforcement learning in the IRAC-MCS model.

Training Phase	Number of Iterations	Average Energy Consumption Ratio (kW·h/m³)	Average Production Efficiency (m/min)	Task Completion Time Reduction (%)	Comprehensive Energy Efficiency Score
Initial Exploration	1,000	1.95 ± 0.12	0.22 ± 0.03	5.3 ± 0.8	75.8 ± 1.5
	1,050	1.92	0.23	5.8	76.5
	1,100	1.90	0.24	6.1	77.2
Average	1,050	1.92	0.23	5.73	76.5
Mid-term Learning	5,000	1.68 ± 0.10	0.25 ± 0.02	12.1 ± 1.5	85.4 ± 1.8
	5,100	1.65	0.26	12.8	86.2
	5,200	1.63	0.27	13.5	87.0
Average	5,100	1.65	0.26	12.8	86.2
Late Convergence	10,000	1.52 ± 0.08	0.28 ± 0.02	18.7 ± 1.2	92.3 ± 1.5
	10,100	1.50	0.29	19.3	92.8
	10,200	1.48	0.30	19.9	93.3
Average	10,100	1.50	0.29	19.3	92.8
Stable Operation	20,000	1.45 ± 0.07	0.30 ± 0.02	25.4 ± 1.5	96.1 ± 1.2
	20,100	1.44	0.31	26.0	96.5
	20,200	1.43	0.32	26.5	96.8
Average	20,100	1.44	0.31	26.0	96.5

In Table 5, during the initial exploration stage, the IRAC-MCS model showed preliminary optimization effects, with an average energy consumption ratio of 1.92 kW·h/m³, a production efficiency of 0.23 m/min, and a comprehensive energy efficiency score of 76.5 points. As the mid-term learning stage progressed, the performance of the research model significantly improved, with an average energy consumption ratio reduced to 1.65kW·h/m³, production efficiency increased to 0.26m/min, task completion time shortened by 12.8%, and comprehensive energy efficiency score jumped to 86.2 points. After entering the later convergence stage, the optimization effect of the model tended to stabilize, and various indicators were further improved. The average energy consumption ratio was reduced to 1.50kW·h/m³, the production efficiency reached 0.29m/min, and the comprehensive energy efficiency score was as high as 92.8 points. In the stable operation phase, the model showed the best performance, with an average energy consumption ratio as low as 1.44kW·h/m³, a production efficiency of 0.31m/min, a task completion time shortened by 26.0%, and a stable comprehensive energy efficiency score of 96.5 points. Overall, it has been verified that the reinforcement learning module can effectively drive long-term self-optimization of excavation strategies, significantly improving the energy efficiency and effectiveness of excavation operations.

### 3.4. Engineering feasibility and pilot deployment case study

To bridge the gap between simulation and engineering application, and verify the adaptability of the IRAC-MCS model in actual underground environments, a specific deployment feasibility analysis and a pilot engineering case plan were developed. (1) Computational Requirements and Underground Adaptability: The complex environment of underground coal mines imposes strict constraints on hardware. The trained PPO network is lightweight, with a single-step inference time of approximately 15ms. It is designed to be deployed on an explosion-proof edge computing terminal (e.g., based on NVIDIA Jetson AGX Orin module, certified for Ex d I Mb). This hardware configuration provides sufficient AI computing power (up to 275 TOPS) to satisfy the real-time control requirement of the roadheader (control cycle typically 50–100ms) while withstanding the high humidity, dust, and vibration typical of the underground working face. (2) Compatibility with Existing Systems: The model adopts a non-intrusive integration strategy to ensure compatibility with existing roadheader control systems. The intelligent decision signals (cutting speed/swing speed) are converted into standard industrial communication protocols: Signal Interface: Converted via D/A modules into 4-20mA analog signals or encapsulated into CAN bus (CANopen/J1939) messages. Logic Interlocking: The output acts as a “suggested value” input to the existing PLC (e.g., Siemens S7-1500), ensuring that the underlying safety logic (such as overload protection) of the original hydraulic system remains the highest priority. (3) Field Engineering Verification Design and Implementation: To validate the fidelity of the IRAC-MCS model in actual engineering environments, a rigorous field verification experiment was designed and conducted in a transport roadway of a high-gas mine in Shanxi Province. The roadway section measures 5.5m × 4.5m and consists of mudstone and sandstone interbeds (f = 6 ~ 8). Experimental Setup: The test platform was based on an EBZ-260 cantilever roadheader. To achieve non-intrusive data acquisition, high-precision Hall effect current sensors were installed in the frequency converter cabinet to monitor the three-phase current of the cutting motor. The data acquisition system utilized a high-performance industrial edge computing terminal connected via the CAN bus, with a sampling frequency set to 50 Hz to capture dynamic load fluctuations.Experimental Protocol: The verification adopted a “Shadow Mode” protocol. In this mode, the roadheader was operated manually by experienced drivers to perform standard cutting cycles (sumping, traversing, and withdrawing). Simultaneously, the IRAC-MCS model ran in parallel on the edge terminal, receiving real-time state data and outputting simulation results without interfering with the actual control loop. Data Processing: The raw field data from a continuous cutting cycle of 20 minutes was extracted. To eliminate high-frequency electromagnetic noise in the industrial environment, a sliding average filter (window size = 5) was applied to the measured signals. The stator current was then converted into cutting torque based on the motor’s characteristic curve. Finally, the processed field data was time-aligned and compared with the simulation results under identical geological parameter settings. The comparison focuses on two key indicators: Mean Load and Load Fluctuation Coefficient. The comparative results are presented in Table 6.

**Table 6:** Comparison between field measured data and simulation results.

Working Condition	Performance Indicator	Simulation Value	Field Measured Value	Relative Error (%)
Mudstone (f = 6)	Mean Load (kN)	185.4	192.6	3.88
	Fluctuation Coeff.	0.24	0.27	/
Sandstone (f = 8)	Mean Load (kN)	245.8	261.3	6.30
	Fluctuation Coeff.	0.31	0.35	/
Mixed Interface	Response Time (s)	0.45	0.52	15.5

As indicated in Table 6, the relative error between the simulated mean load and the field measured data was controlled within 6.5%. Although the field measured fluctuation coefficient was slightly higher than the simulation value due to the vibration of the machine body and external noise, the overall load trend remained consistent. The response time of 0.52s recorded in the field environment further confirms that the proposed control strategy meets the real-time requirements of industrial deployment. This preliminary verification demonstrates that the IRAC-MCS model possesses high engineering applicability.

## 4. Conclusion

Aiming at the severe load fluctuations and frequent pick failures caused by complex coal-rock geology in the cutting head of coal mine roadheaders, and the difficulty of accurately capturing micro loads in existing macroscopic control, a multi-scale coupled simulation RBF-PID adaptive cutting head load intelligent control model, namely IRAC-MCS model, was constructed. The study aimed to improve the accuracy of micro force prediction by modifying the classical load model through FEM, and combining it with the NSGA-II algorithm to achieve multi-objective optimization of cutting parameters. The RBF-PID controller was used to dynamically adjust parameters, and the MBD-DEM and PPO extended model were introduced to enhance the adaptability to complex working conditions. The performance verification was carried out from multiple operating conditions. Under constant operating conditions, the MSE of the IRAC-MCS model was only 15, the control overshoot was 0, and the steady-state simulation accuracy was excellent. When there was a slight change in coal-rock hardness, a warning could be given 3.5 seconds in advance, and the prediction error of the peak load was only 4.2%, demonstrating outstanding warning and prediction capabilities. Under single-point fracture failure, its recovery time was 2.1 seconds, which could efficiently respond to the fault. In the case of sudden load conditions, the normalized indicators of response time, peak overshoot, and recovery time of the model were 10%, 0%, and 15%, respectively, and the dynamic response was rapid and stable. When the noise intensity was 0.8, its MSE was about 200, indicating strong anti-interference ability. The simulation test also showed excellent performance, with a path planning error of 0.05m for regular roadways, with high roadway accuracy. In long-term operation, the energy efficiency was improved by 15.3%, with a stable energy consumption ratio of 1.44 kWh/m³ and a 26.0% reduction in task completion time. Energy efficiency and operational efficiency have been optimized in a dual manner. In summary, the research has verified the advantages of the IRAC-MCS model in load simulation accuracy, control stability, and adaptability to complex working conditions, which can effectively reduce the pick failure and improve excavation efficiency. However, limitations still exist. While the pilot study confirmed the feasibility of signal acquisition, ensuring long-term sensor stability under extreme geological conditions requires further optimization. Additionally, the current MBD-DEM module needs enhancement to better simulate macroscopic crack propagation in highly fractured coal-rock. Future work will focus on two aspects to improve engineering universality: (1) Theoretical Optimization for Special Geology: To adapt to high-gas and fractured scenarios, the coal-rock constitutive model will be upgraded by introducing fracture mechanics and gas-solid coupling equations, specifically enhancing the simulation fidelity of crack propagation and gas leakage inducement mechanisms. (2) Engineering Solution for Sensor Integration: To overcome the obstacles of installing contact sensors on unstable, multi-layered, and highly fragmented rock surfaces, a non-invasive multi-source fusion perception strategy will be adopted. Instead of relying on rock-mounted sensors, the system will fuse machine-side internal response signals (e.g., stator current, cylinder pressure) with non-contact external perception (e.g., explosion-proof LIDAR or structured light cameras). Furthermore, robust filtering algorithms based on deep denoising auto-encoders will be developed to isolate effective load features from the high-noise environment caused by severe vibration and dust.

## Supporting information

S1 File Minimal data set definition. (DOCX)
