# Supplementary material for: Dynamic simulation and intelligent control technology for cutting head load of coal mine roadheader
Source: PLoS One. 2026 Mar 9;21(3):e0343250. doi: 10.1371/journal.pone.0343250 (PMC12970920; doi:10.1371/journal.pone.0343250)
Supplement: S1 File — (DOCX) [file pone.0343250.s001.docx]

**The data in Figure 9(a)**

| Time (s) | IRAC-MCS Load (kN) | PID Load (kN) | B-SMC Load (kN) | RBFNN Load (kN) |
| --- | --- | --- | --- | --- |
| 0 | 2900 | 2900 | 2900 | 2900 |
| 2 | 2985 | 3150 | 3050 | 2990 |
| 5 | 3000 | 3420 | 3180 | 3040 |
| 8 | 3000.2 | 3500 | 3200 | 3050 |
| 10 | 3000.05 | 3650 | 3200 | 3100 |
| 15 | 3000 | 3250 | 3100 | 3010 |
| 20 | 3000.02 | 3380 | 3150 | 3050 |
| 30 | 3000 | 3200 | 3100 | 3020 |
| 40 | 3000 | 3120 | 3050 | 3010 |
| 50 | 3000 | 3080 | 3025 | 3005 |
| 60 | 3000 | 3050 | 3015 | 3002 |
| 70 | 3000 | 3030 | 3010 | 3001 |
| 80 | 3000 | 3020 | 3005 | 3000.5 |
| 90 | 3000 | 3010 | 3002 | 3000 |
| 100 | 3000 | 3005 | 3000 | 3000 |

**The data in Figure 9(b)**

| Model Type | MSE | Control Overshoot (%) |
| --- | --- | --- |
| IRAC-MCS | 15 | 0 |
| PID | 350 | 16.7 |
| B-SMC | 120 | 6.7 |
| RBFNN | 40 | 1.7 |

**The data in Figure 10(a)**

| Time (s) | IRAC-MCS Load (kN) | PID Load (kN) | B-SMC Load (kN) | RBFNN Load (kN) |
| --- | --- | --- | --- | --- |
| 0 | 3000.0 | 3000.0 | 3000.0 | 3000.0 |
| 6 | 3002.1 | 3001.5 | 3003.2 | 3001.8 |
| 12 | 3005.4 | 3450.2 | 3210.5 | 3120.8 |
| 18 | 3001.2 | 3200.6 | 3100.4 | 3050.3 |
| 24 | 3000.5 | 3100.3 | 3050.1 | 3020.7 |
| 30 | 3000.1 | 3050.8 | 3020.5 | 3010.2 |

**The data in Figure 10(b)**

| Model Type | Response Time (%) | Peak Overshoot (%) | Recovery Time (%) |
| --- | --- | --- | --- |
| IRAC-MCS | 10 | 0 | 15 |
| PID | 80 | 16.7 | 90 |
| B-SMC | 40 | 6.7 | 45 |
| RBFNN | 25 | 1.7 | 35 |

**The data in Figure 11**

| Noise Intensity | IRAC-MCS MSE | PID MSE | B-SMC MSE | RBFNN MSE |
| --- | --- | --- | --- | --- |
| 0 | 15 | 350 | 120 | 40 |
| 0.2 | 50 | 600 | 300 | 150 |
| 0.4 | 100 | 900 | 600 | 300 |
| 0.6 | 150 | 1200 | 900 | 450 |
| 0.8 | 200 | 1550 | 1200 | 600 |
| 1 | 220 | 1800 | 1500 | 750 |

**The data in Figure 12**

| Training Iteration | IRAC-MCS MSE | RBFNN MSE |
| --- | --- | --- |
| 0 | 30 | 60 |
| 200 | 15 | 40 |
| 400 | 10 | 35 |
| 600 | 8 | 32 |
| 800 | 8 | 31 |
| 1000 | 8 | 31 |

**The data in Figure 13**

| Roadway Type | Path Planning Error (m) | Load Fluctuation Rate (%) | Average Cutting Tooth Wear Rate (%) | Simulation Running Time (min) |
| --- | --- | --- | --- | --- |
| Regular roadway | 0.05 | 2.07 | 5.77 | 45 |
| Inclined roadway | 0.08 | 3.1 | 6.5 | 50 |
| Curved roadway | 0.12 | 4.2 | 7.8 | 60 |
| Roadway with interbedded gangue | 0.15 | 4.8 | 9.3 | 75 |
